# Channel Waveguides in Lithium Niobate and Lithium Tantalate

**DOI:** 10.3390/molecules25173925

**Published:** 2020-08-27

**Authors:** Yi Lu, Benjamin Johnston, Peter Dekker, Michael J. Withford, Judith M. Dawes

**Affiliations:** MQ Photonics, Department of Physics, Macquarie University, Sydney, NSW 2109, Australia; coeus.lu@gmail.com (Y.L.); Benjamin.Johnston@mq.edu.au (B.J.); peter.dekker@mq.edu.au (P.D.); michael.withford@mq.edu.au (M.J.W.)

**Keywords:** lithium niobate, laser processing, crystalline waveguide, liquid phase epitaxy

## Abstract

Low-loss photonic waveguides in lithium niobate offer versatile functionality as nonlinear frequency converters, switches, and modulators for integrated optics. Combining the flexibility of laser processing with liquid phase epitaxy we have fabricated and characterized lithium niobate channel waveguides on lithium niobate and lithium tantalate. We used liquid phase epitaxy with K_2_O flux on laser-machined lithium niobate and lithium tantalate substrates. The laser-driven rapid-prototyping technique can be programmed to give machined features of various sizes, and liquid phase epitaxy produces high quality single-crystal, lithium niobate channels. The surface roughness of the lithium niobate channels on a lithium tantalate substrate was measured to be 90 nm. The lithium niobate channel waveguides exhibit propagation losses of 0.26 ± 0.04 dB/mm at a wavelength of 633 nm. Second harmonic generation at 980 nm was demonstrated using the channel waveguides, indicating that these waveguides retain their nonlinear optical properties.

## 1. Introduction

Integrated optics employs a wide range of miniaturized, high-speed, broad-band, and reliable components suited for telecommunications, data processing, optical computing and other applications. Because of its excellent electro-optic and nonlinear properties, lithium niobate (LiNbO_3_) is a key material for active optical waveguide applications [1,2,3,4,5].

Channel waveguides, the basic building blocks of optical circuits used in both active and passive devices of integrated optics, are fabricated by many methods. Proton exchange waveguides [6] offer high refractive index contrast in one polarization, and this technique has been employed for precision structures such as tapers [7]. Ion implantation of titanium or zinc into lithium niobate is a well-established technique for creating waveguides, but the dopant profile is sensitive to annealing [2,8] and can be difficult to control. The dopant ions can also be substituted into the epitaxially-grown waveguides during the growth phase [9]. Direct laser treatment of ion-doped lithium niobate can induce gratings and channel optical waveguides within the surface layers [10]. Alternatively thin films of LiNbO_3_ deposited on sapphire can be ablated by an excimer laser to create smooth ablated structures, with little damage to adjacent areas [11]. Recently rare-earth-ion-doped lithium niobate waveguides have been fabricated for on-chip integrated structures [12,13].

Ridge waveguide structures can be fabricated in planar lithium niobate devices by plasma dry etching [14,15], wet etching [16], or ion beam etching [17], and also precision diamond cutting [18]. Alternatively, femtosecond laser machining is increasingly favored for creation of ridge optical waveguides in various crystalline laser materials [19,20]. The femtosecond laser direct writing technique has been demonstrated for the fabrication of Type I and Type II waveguides and depressed cladding waveguides in lithium niobate, for efficient nonlinear frequency conversion [21,22,23]. Laser direct writing can also facilitate hybrid integrated structures [24]. Ridge waveguide lasers were fabricated in neodymium doped lithium niobate via femtosecond direct laser writing of gradient index planar waveguides, fabricated by Zn in-diffusion [25]. The ridge waveguide approach increases the index contrast, thus increasing the mode confinement for small bend radius waveguide features.

The crystal growth process chosen can ensure high quality crystallinity in the lithium niobate. Low-loss thin films of lithium niobate have been sputtered onto alternative substrates including sapphire and silica [26]. Liquid phase epitaxial growth on patterned substrates also offers flexible options for fabricating waveguides [27,28]. Here we report liquid phase epitaxy growth of channel waveguides on laser-machined substrates to fabricate high optical quality lithium niobate channel waveguides with single crystalline properties on lithium niobate and lithium tantalate substrates. Liquid phase epitaxy is a versatile crystal growth technique which creates a step-index profile rather than a graded-index profile for improved mode confinement. A slight dissolution at the surface during the process leads to smoothing of the waveguide edges, thus reducing the losses. Furthermore, by using lithium tantalate substrates, high refractive index contrast can be realized for both optical polarization directions.

## 2. Results and Discussion

### 2.1. Laser Processing

Laser machining of the LiNbO_3_ and LiTaO_3_ substrates was carried out using a femtosecond pulsed Ti:sapphire laser. The laser-machined features were studied in detail with different sample feed-rates and numbers of passes. The shape of the machined features is also governed by the laser beam profile. The laser beam used in micro-machining has a typical Gaussian profile which can be expressed by:

(1) I(r)=I(0) e−2rw02

where *I*(*r*) is the local irradiance, *I (0)* is the peak irradiance, *w_0_* is the radius where the irradiance has decreased to 1/e^2^ of the peak. The Gaussian irradiance profile needs to be considered when translating pulse energy to the irradiance or energy distribution on target. Laser ablation occurs when the irradiance exceeds the ablation threshold, but not all the beam profile lies above this threshold. The laser-machined feature diameter *D* can be related to the Gaussian beam profile and irradiance threshold by:

(2) $$D^{2}=2w_{0}^{2}\ln\frac{I_{peak}}{I_{threshhold}}$$

**Figure 1:**
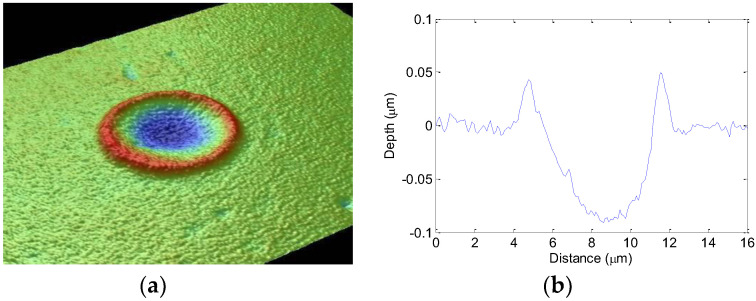
(**a**) 3D visualization of a single shot ablation crater from optical profilometry. (**b**) 2D cross-sectional data indicating the diameter and depth of the crater (Veeco NT3300, Bruker Nano Surface, Tucson, AZ, USA).

Experimentally this relationship is used to determine the beam waist on target and the pulse energy required for ablation from the slope (*2w*_0_^2^) and intercept (ln*(I_threshold_)*) respectively, of Equation (2). Measurements of the “single shot” crater diameters and depths for various pulse energies were carried out using optical profilometry (Veeco NT3300, Bruker Nano Surface, Tucson, AZ, USA). A typical optical profile measurement is shown in Figure 1 with both a 3D visualization and 2D cross-sectional representation of the data.

**Figure 2:**
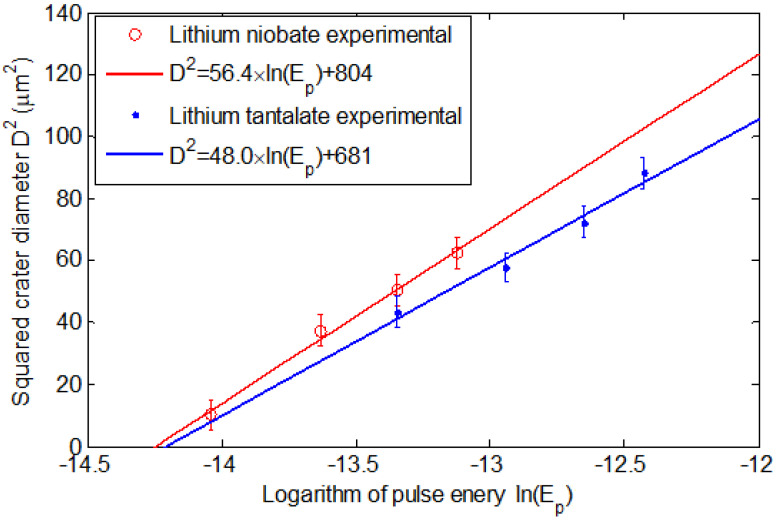
Evolution of the square of the crater diameter in relation to the logarithm of pulse energy, consistent with Equation (1).

The evolution of the crater diameter as the pulse energy is varied near the ablation threshold is shown for both lithium niobate and lithium tantalate in Figure 2, with the square of the diameter plotted against the logarithm of the pulse energy plotted consistent with Equation (2). The experimental data suggest a beam waist of *w_0_* ≈ 4.8–5 μm (1/e^2^ diameter of 9.6–10 μm) and corresponding threshold fluence of 1.46 J/cm^2^ for lithium niobate and 1.80 J/cm^2^ for lithium tantalate, which is consistent with the results of Zhang et al. [28].

**Figure 3:**
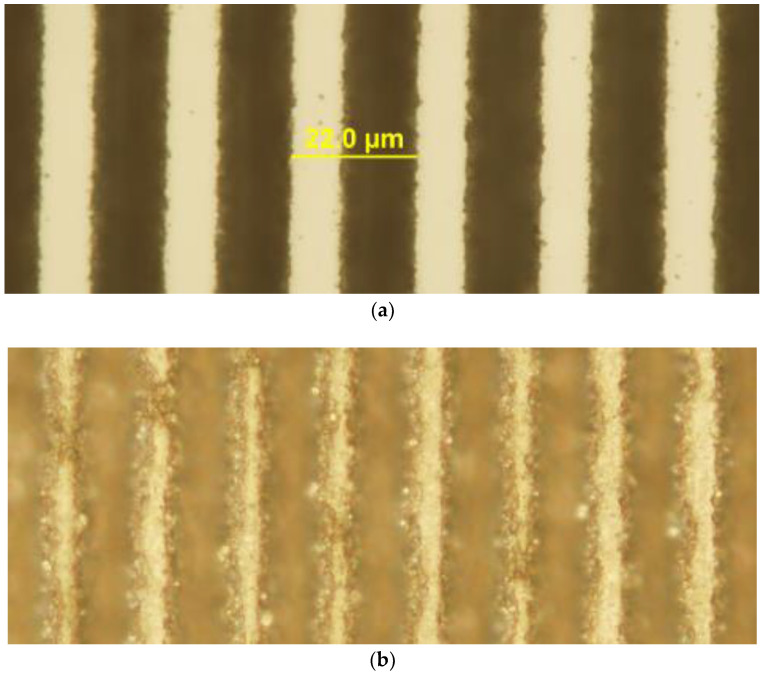
(**a**) Trenches machined in lithium niobate with 6.2 J/cm^2^. The period of the trenches is 22.0 μm as shown on the scale bar. (**b**) Trenches machined in lithium niobate with 9.5 J/cm^2^.

The fluence appropriate for producing high quality trenches on the crystal surface was determined empirically by visual inspection of the trenches after laser machining. It was found that a 2 mW average power, corresponding to a fluence of ~6.2 J/cm^2^ was sufficient to produce high quality features. Increasing the power to 3 mW (9.5 J/cm^2^) had an adverse effect on the quality of features with a marked increase in edge chipping and debris. Optical micrographs (Olympus BX 61, Sydney, Australia) of typical arrays of trenches machined at these two fluences are shown for comparison in Figure 3.

**Figure 4:**
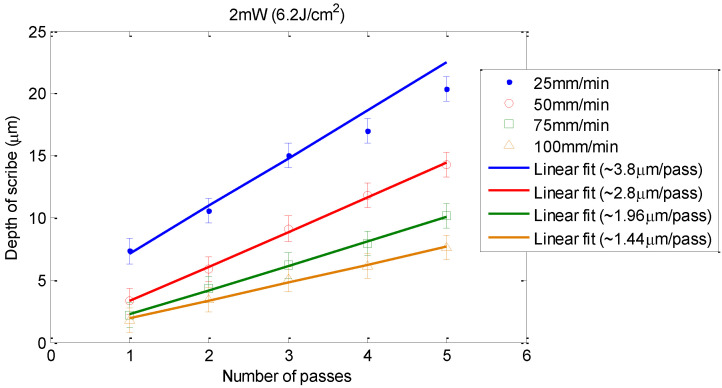
Measured trench depths for one to five passes over a range of translation speeds.

Control over the trench depth was exercised by varying the sample translation speed through the laser beam and by varying the number of passes. The measured depths (from the microscope images of the trench cross-sections) for one to five passes over a range of translation speeds from 25 mm/min to 100 mm/min are shown in Figure 4. The trench depth generally shows a linear relationship with respect to the number of passes.

**Figure 5:**
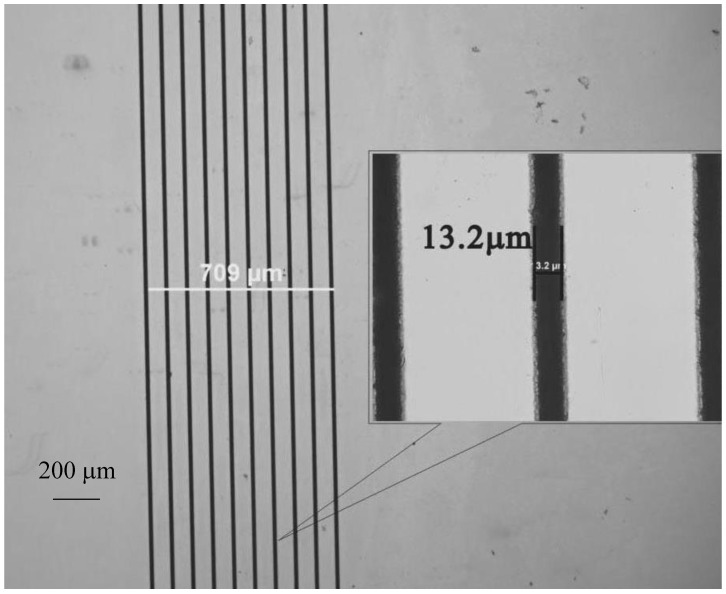
Optical micrograph of laser micro-machined features on a lithium niobate substrate. The distance across all the trenches is 709 μm and the width of a single trench is 13.2 μm. The 200 μm scale bar is shown.

Microscope images (Olympus BX 61, Sydney Australia) of the machined lithium niobate substrates before the liquid phase epitaxy experiments were recorded to compare with later results after crystal growth. A typical sample is shown in Figure 5. The microscope images show the machined lithium niobate substrate samples with machined trenches of width about 13.2 μm and spacing from 5 μm up to 100 μm. All these features were pre-programmed and are adjustable.

### 2.2. Liquid Phase Epitaxial Crystal Growth

**Figure 6:**
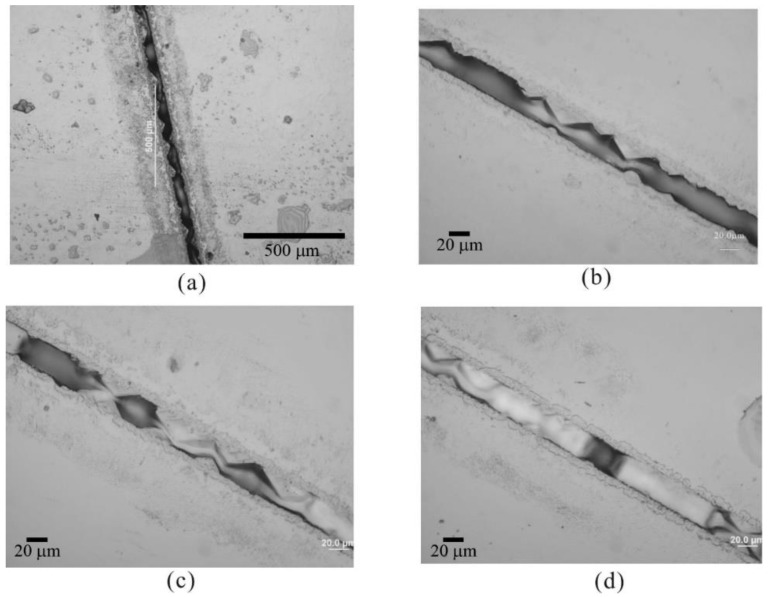
(**a**–**d**) Optical microscope images of consecutive stages of liquid phase epitaxy growth (up to 10 min) inside V grooves on lithium niobate substrates.

**Figure 7:**
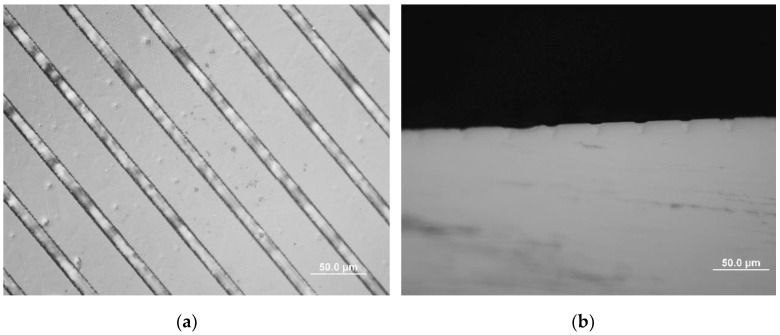
Optical micrographs showing an example of liquid phase epitaxial growth of lithium niobate on a lithium tantalate substrate at a temperature 0.5 °C below saturation temperature for ten minutes (**a**) top view; (**b**) end view.

The channels were filled by liquid phase epitaxial growth with K_2_O flux using established thin film growth conditions [29]. As indicated in Figure 6a, the growth initiated in both sidewalls of the V-groove and each nucleation site has the shape of a pyramid. Lithium niobate grown in K_2_O flux has strong faceting effects along the (012) direction [30]. The pyramids inside the V groove were most likely caused by this faceting tendency. The pyramids continue growing at both sidewalls of the V groove in Figure 6b and join together at Figure 6c. In Figure 6d, isolated islands from different nucleation sites start to join together within the V groove. No apparent growth was observed on the planar surface outside the V groove. On the contrary, some degree of dissolution occurred along the edge of the V groove. Similar growth surface morphology was reported in an epitaxial growth model on patterned substrates of GaAs (001) surface and (111) V groove [31]. Figure 7 shows microscope images of an as-grown machined sample of lithium niobate waveguides grown on a lithium tantalate substrate. For good control of the surface quality, the growth rate needs to be stable to avoid poly-crystallization, kinetic roughening, and mis-orientation roughening. To achieve this, we set the growth temperature to be 0.5 °C below the saturation temperature and set the growth time to be 10 min. As indicated in Figure 7 (lithium niobate channel waveguides grown on a machined lithium tantalate substrate), the crystallization only took place inside the V grooves while leaving the plane surface untouched, which gave us much better surface quality.

**Figure 8:**
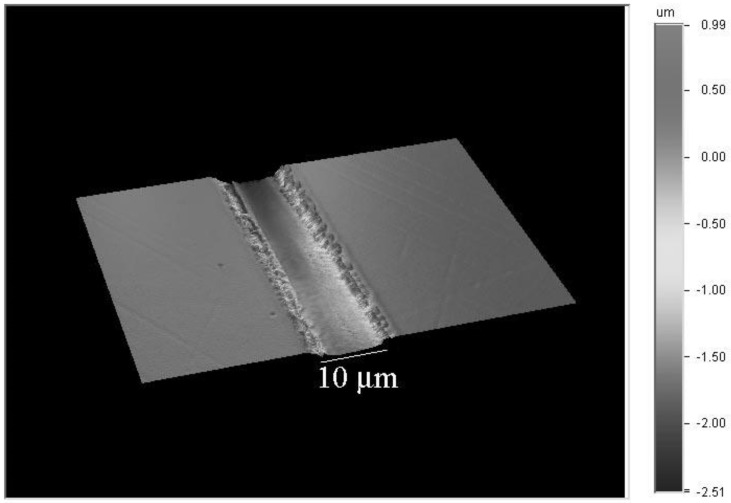
Three dimensional surface image of the as-grown liquid phase epitaxial layer on the machined lithium tantalate substrate from the optical profiler (Veeco, Bruker Nano Surface, Tucson, AZ, USA).

The 3-D contour map of the liquid phase epitaxy grown sample surface is shown in Figure 8. The surface roughness inside the V groove was checked with an optical profiler (Veeco, Bruker Nano Surface, Tucson, AZ, USA). The surface roughness of the plane surface of lithium tantalate substrate (8.6 nm) was unchanged after liquid phase epitaxial thin film growth, while the surface roughness inside the machined V groove was ~92 nm. The surface roughness of the as-grown lithium niobate channels was larger than that for waveguides fabricated by the titanium in-diffusion technique, which is 20–62 nm [32]. Further optimization of liquid phase epitaxial growth parameters is required to improve the surface roughness of the channel waveguides.

### 2.3. Optical Characterization

**Figure 9:**
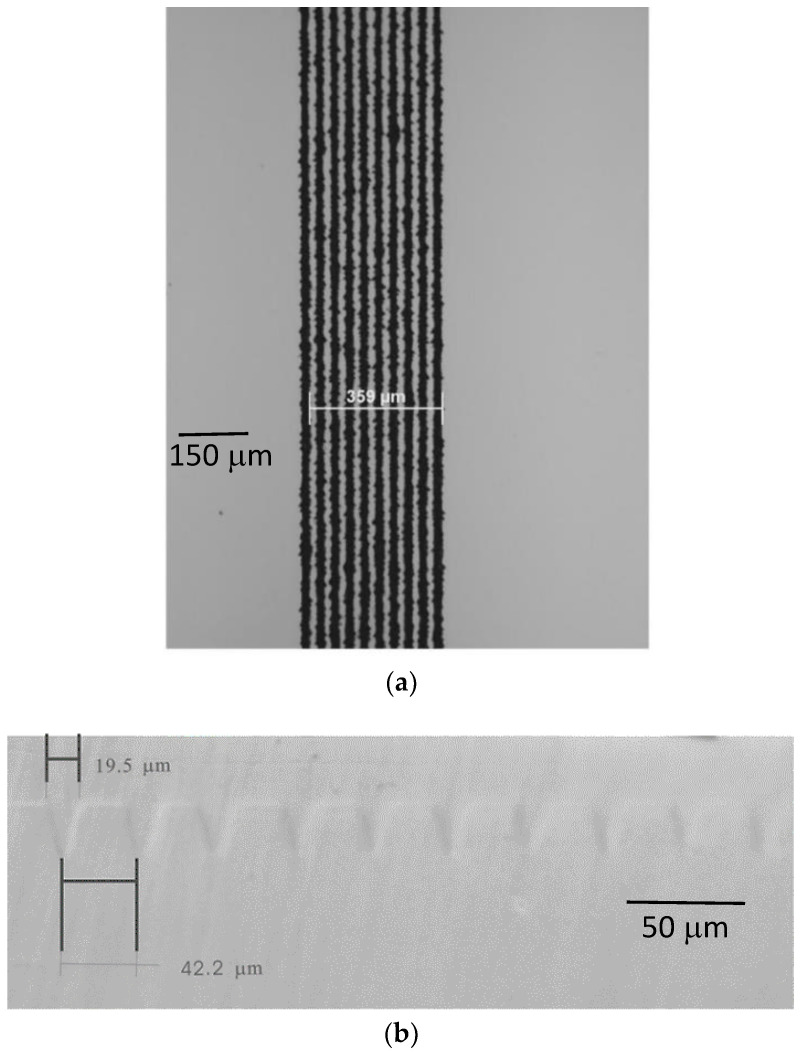
(**a**) Top view of machined lithium niobate substrate. Scale bar of 150 μm is shown. (**b**) Differential interference microscopic image of the polished end face of as-grown lithium niobate channel waveguides after liquid phase epitaxial thin film growth. Scale bar of 50 μm is shown.

Optical micrographs of lithium niobate channel waveguides on lithium niobate substrates are shown in Figure 9. Figure 9a shows the top view of the machined lithium niobate substrate. The end faces of the as-grown channel sample were polished, as shown in Figure 9b. The top surface of this sample was lightly polished after liquid phase epitaxial thin film growth. The as-grown channels were observed using the microscope in difference interference contrast mode. The average width of the V groove channels was about 20 μm while the average spacing between the channels was about 46 μm as determined by the initial laser machining.

**Figure 10:**
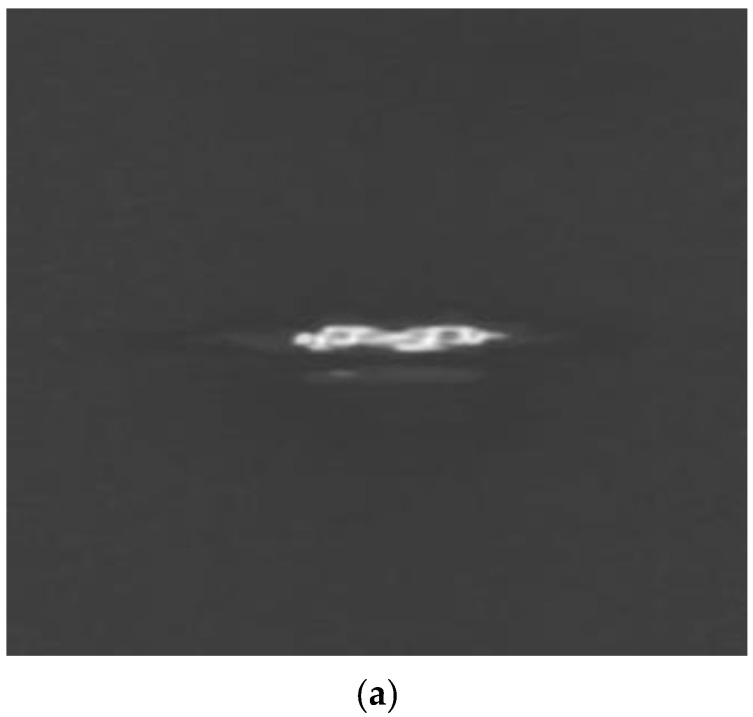

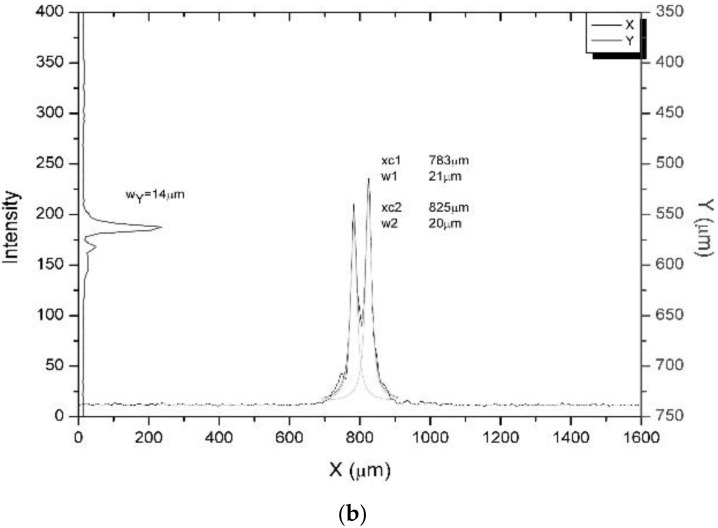
(**a**) Near-field beam profile and (**b**) mode cross-section for TE modes propagating at 1064 nm in 20 μm lithium niobate channel waveguides on lithium tantalate samples obtained by imaging with a 10× microscope objective. (Spiricon, Logan, UT, USA).

More detailed optical characterization and simulations of the lithium niobate channel waveguides on lithium tantalate substrates were carried out. The waveguiding properties within the channel waveguides, were characterized by coupling a probe beam into the guide and imaging the near-field beam profiles by a CCD camera. We used a linearly polarized Nd:YAB laser probe beam operating at 1064 nm. The input beam was focused down to 50 μm using a 10× microscope objective on the end face of the channel waveguide sample. The near-field of the output TE beam profile was collected by a 4× microscope objective and imaged using a TM-745 CCD camera (Pulnix, Sunnyvale, CA, USA). The near-field beam profiles were analyzed by an optical beam profiler (Spiricon, Logan, UT, USA) and are shown in Figure 10. The TM modes are not supported in this sample with lithium niobate as a substrate.

As shown in the image, the beam has two V shaped profiles with beam radius about 20 μm in the X direction and 14 μm in the Y direction. The beam shape and size are comparable with the machined features. The input laser beam was coupled into two channel waveguides. The spacing between the two beams is 42 μm which is also the same as the spacing between the machined V grooves.

**Figure 11:**
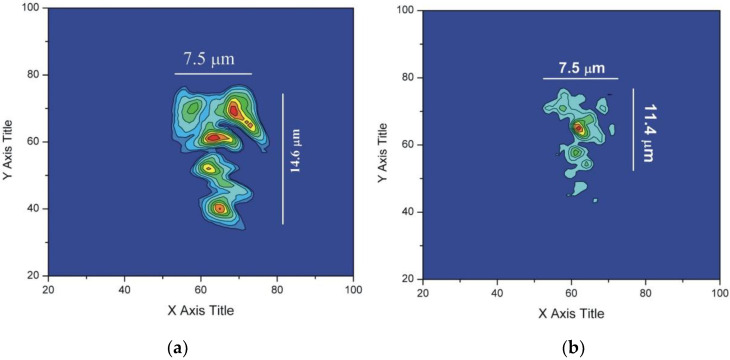
Near-field beam profiles for (**a**) 633 nm and (**b**) 975 nm from a 7 μm lithium niobate channel with lithium tantalate as the substrate; imaging with a 40× microscope objective.

The near-field beam profiles indicate well-confined waveguide structures. The near-field profiles from the channel waveguide sample were characterized by end-fire coupling of 975 nm and 633 nm light from single mode optical fiber. The output near-field profiles imaged by a 40× microscope objective are shown in Figure 11.

**Figure 12:**
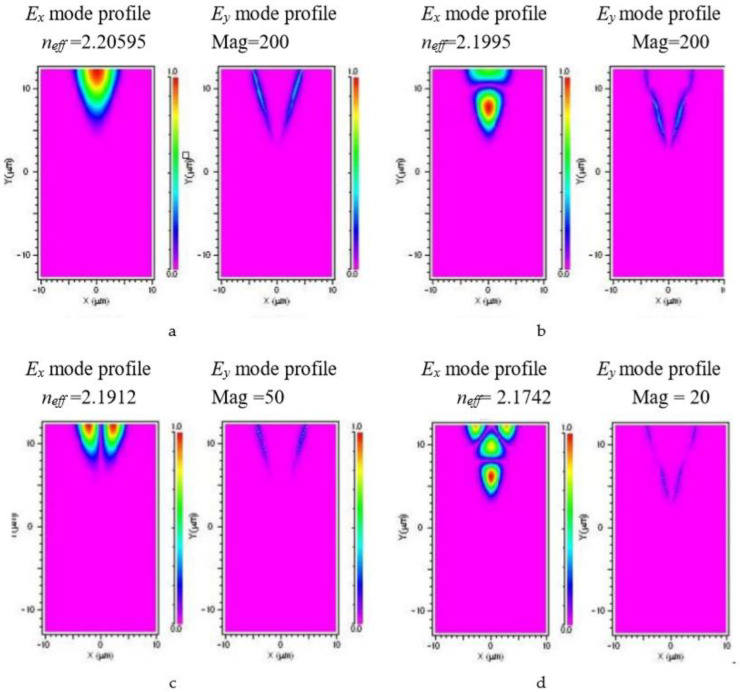
(**a**–**d**). Calculated *E_x_* and *E_y_* mode profiles for lithium niobate channel waveguides on lithium tantalate substrates showing that the y-component of the electric field is negligible. The *E_y_* mode profile is proportionally scaled for each profile by (**a**) 200×, (**b**) 100×, (**c**) 50×, and (**d**) 20× magnification respectively. The vertical and horizontal axes are marked in μm. The *n_eff_* values are: (**a**) 2.2059, (**b**) 2.1995, (**c**) 2.1912, and (**d**) 2.1742 respectively.

The near-field beam profiles indicate multimode propagation within the channel, [33] which is consistent with the refractive index contrast (*Δn_o_* ~ 0.1) provided by the lithium tantalate substrate and lithium niobate channels. Numerical simulations were performed using BeamPROP (part of the RSoft Photonics CAD Suite, USA) which is based on a finite difference beam propagation method assuming 10 nm step sizes in the *x*, *y* and *z* directions. The simulated TE mode profiles are plotted in Figure 12. The simulation is based on the channel size measured by the microscope images and the refractive index for bulk lithium tantalate and lithium niobate at a wavelength of 0.980 μm for TE mode propagation. The refractive indices of lithium niobate (*n_o_* = 2.2355 and *n_e_* = 2.1480) [34] and lithium tantalate (*n_o_* = 2.140 and *n_e_* = 2.139) [35] were calculated from the Sellmeier equations, which depend on temperature, wavelength and lithium concentration. The calculated mode profiles have similar size to the measured beam profiles and are also multimode.

**Figure 13:**
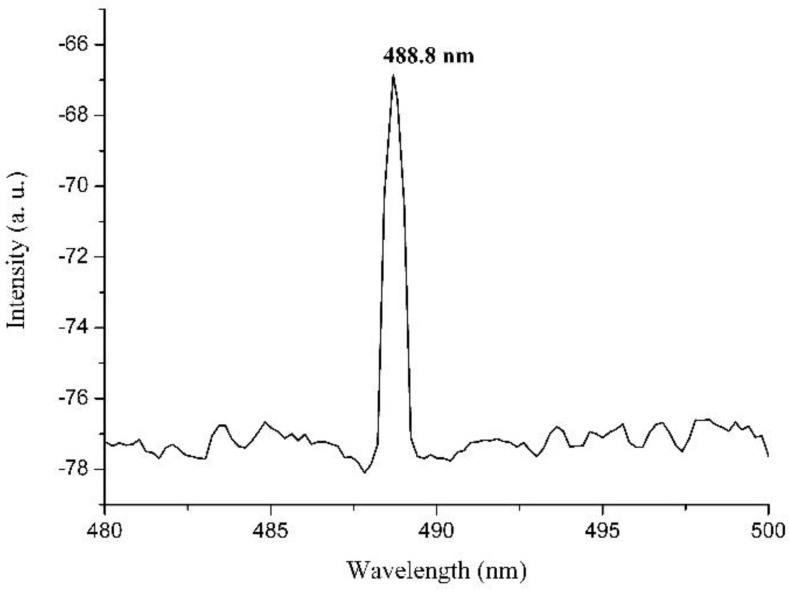
Second harmonic generation spectrum detected from lithium niobate channel waveguides with lithium tantalate substrate.

Temperature-tuned second harmonic generation of a 975 nm diode laser was also measured from the lithium niobate channel waveguides. The second harmonic generation phase-matching temperature for stoichiometric lithium niobate is 42 °C for 979 nm fundamental light, and blue second harmonic light is detectable from the channel samples at room temperature, indicating the good crystallinity of the channel waveguide. The second harmonic output spectrum is plotted in Figure 13.

The propagation loss of the as-grown channel sample was measured by the back-reflection method at 633 nm (See Section 4). The propagation loss was 0.26 ± 0.04 dB/mm. The relatively high propagation loss within the channel samples is attributed to the interaction of higher order modes with the surface roughness of the lithium niobate channel.

## 3. Conclusions

Liquid phase epitaxial growth on laser micro-machined lithium tantalate and lithium niobate substrates is a practical and promising way to fabricate channel waveguides, and allows rapid prototyping of the guide design to be implemented. Lithium niobate and lithium tantalate substrates were first patterned by laser micro-machining. The fabrication of desired features is programmable with prescribed parameters such as pulse energy, the number of passes on the same area, the feed-rate of the sample with respect to the laser spot and the laser beam profile. After liquid phase epitaxy growth of lithium niobate on the patterned substrate, the surface roughness was measured to be ~92 nm, compared with ~60 nm for channel waveguides fabricated by the ion in-diffusion technique. In the case of liquid phase epitaxial growth, the waveguide is inherently crystalline and of good optical quality as evidenced by the second harmonic generation results and previous studies of liquid phase epitaxially grown planar crystalline waveguides [29].

The beam confinement of the lithium niobate channel waveguide grown on lithium tantalate was examined by propagating a linearly-polarized Nd:YAB probe beam. The output beam, analyzed by a beam profiler, showed coupling into one or two channels, with a spacing of 42 μm. The near-field beam profiles have typical “V” shapes with beam width about 20 μm in the X direction and 14 μm in the Y direction. The near-field beam profile was further examined using a high magnification objective with a single mode fiber probe at 975 nm and 633 nm. The beam profile indicates multi-mode propagation within the channel sample. The beam profiles are consistent with the results of RSoft modelling, providing evidence for the high refractive index contrast (~0.1) in the as-grown channel samples. Second harmonic generation from the channel samples was demonstrated from a fundamental wavelength of 975 nm, indicating the crystal quality of the channel waveguide. The propagation loss was 0.26 ± 0.04 dB/mm, measured by the back-reflection method at 633 nm.

## 4. Materials and Methods

### 4.1. Femtosecond Laser Machining of Substrates

**Figure 14:**
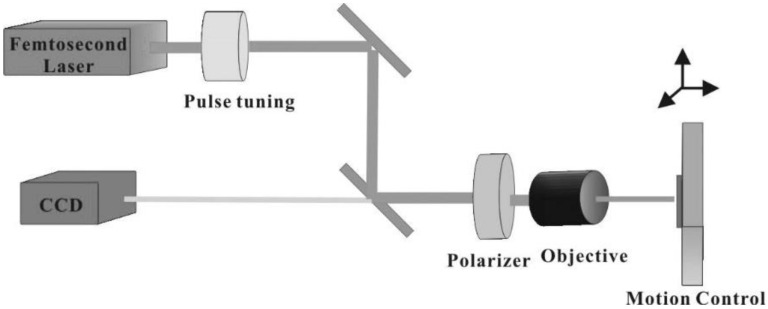
Experiment setup for laser machining.

Laser machining of the LiNbO_3_ and LiTaO_3_ substrates was carried out using a femtosecond laser micromachining facility (OptoFab, Australian National Fabrication Facility), which incorporates an infrared femtosecond pulsed laser (Spectra Physics Hurricane, Spectra Physics, Myrtle Bank, SA, Australia), 3D motion control stages (Aerotech, Inc, Pittsburgh, PA, USA) and diagnostic and alignment tools to create an integrated and completely automated experimental setup for fabricating optical devices. The experimental setup for laser machining is shown in Figure 14.

The Spectra-Physics Hurricane comprises the Mai-Tai Ti:Sapphire 80 MHz oscillator with a 1 kHz regenerative amplifier. The system produces ~100 fs pulse-width pulses with up to 1 mJ energy with a default 1 kHz repetition rate pulse train which may be reduced or controlled externally. The power on target is controlled by a half-wave-plate-linear-polarizer arrangement with the half-wave plate held in a computer-controlled rotation mount for power control. The pulse duration can be controlled by tuning the compression stage gratings.

To fabricate lithium niobate channel waveguides, we used laser micro-machining to write a pattern on the surface of the substrates. The pattern described here was fabricated by moving the substrate through the focus of the laser beam. For example, laser-ablated trenches on the surface of the substrates were attained by moving the sample several passes through the laser focus spot. The depth of the laser-cut features was controlled by the pulse energy, the number of passes on the same area and the feed-rate of the sample with respect to the laser spot. The effect of the pulse energy was studied by comparing the crater morphologies produced by single shot ablation on the substrate materials for various laser parameters. The appropriate laser pulse energy generates smooth craters with little debris deposited on the surrounding surface. To determine the effects of number of passes and sample feeding rate, ideally, we assume the depth of the machined features linearly scales with the number of passes and scales inversely with sample feeding rate. However deviations may arise from changing surface morphology and material properties in the laser-treated area.

### 4.2. Channel Waveguides Grown by Liquid Phase Epitaxy on Laser-Machined Substrates

Liquid phase epitaxy growth was carried out on the laser micro-machined substrates. The experimental setup and procedure were similar to that reported previously for planar samples [29]. Briefly, we used a K_2_O (13.8 mol%) Li_2_O (43.1 mol%) Nb_2_O_5_ (43.1 mol%) flux, at a temperature of 1100 °C and with z-cut lithium niobate or lithium tantalate substrates. The laser-machined substrate was dipped into the preheated molten solution just below the saturation temperature for about 10 min. The growth rate was approximately 4.5 μm/min. Whereas the fractions of Li_2_O and Nb_2_O_5_ in the liquid phase epitaxy growth mixture are equal, the Li/Nb ratio in congruent lithium niobate is 48.6/51.4. We tried to reduce the effect of lithium volatilization on the resulting crystal by rapid growth runs.

The changed surface morphology of the substrate due to laser machining requires more stringent growth controls. The features fabricated by laser machining induce an inhomogeneous morphology on the sample surface at the initial stage of thin film growth. For the z-cut lithium niobate substrate, the surface is the (001) plane while the sidewall inside the machined “V” groove has a different orientation. The initiation of growth during liquid-phase epitaxy takes place in isolated islands [36]. The islands grow and join up to give a smooth surface. For liquid phase epitaxy growth on a patterned surface, the site environment inside the V-groove is different from that of the planar surface, which results in different diffusion rates and growth rates. The isolated islands developing from different sites show steps at the boundaries of adjacent islands.

**Figure 15:**
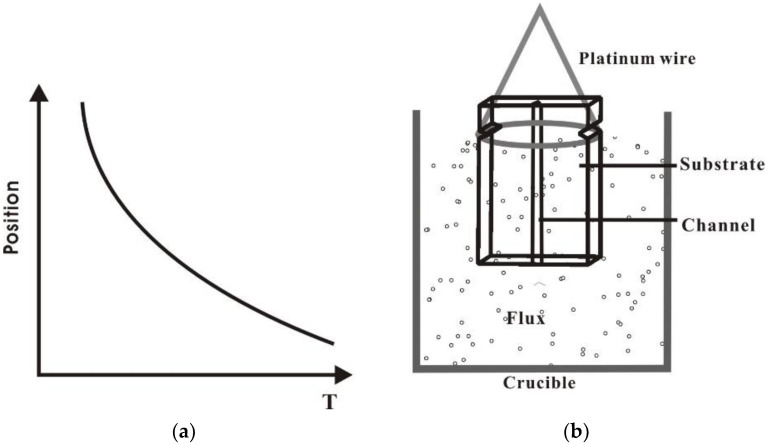
(**a**) Temperature profile in the crucible as a function of position. (**b**) Schematic of the crystal growth furnace layout for liquid phase epitaxial growth of channel waveguides.

The roughness of the epitaxial layer grown on the machined samples arises from two causes. The first is kinetic roughness, which arises from a high deposition rate occurring at the low growth temperature. The second is substrate mis-orientation inside the machined sidewall. The sample was dipped vertically into the LiNbO_3_ flux (see Figure 15b) at the saturation temperature for ten minutes with the machined features aligned vertically inside the furnace. The temperature profile shown in Figure 15a was obtained by suspending a thermocouple inside the empty crucible at different positions.

**Figure 16:**
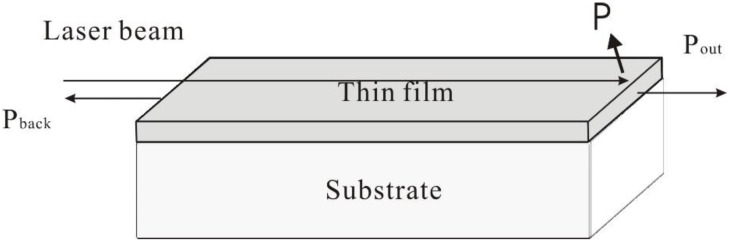
Schematic of the back-reflection method.

The operational principle of the back-reflection method is sketched in Figure 16. The input power, back-reflected power and output power were measured by coupling the light with a short focal length lens from the laser source, into and out of the waveguide, and measuring the power with a standard laser power meter (Newport, Irvine, CA, USA). The back-reflected power was determined by inserting a beam splitter into the path. The propagation loss was extracted from the ratio between the output signal power (P_out_) and the back-reflected signal power (P_back_) [37] where additional losses are represented by P.
